# Light triggered encapsulation and release of C_60_ with a photoswitchable TPE-based supramolecular tweezers

**DOI:** 10.1038/s41598-019-46242-4

**Published:** 2019-07-04

**Authors:** Mousumi Samanta, Anushri Rananaware, Dinesh N. Nadimetla, Sk. Atiur Rahaman, Monochura Saha, Ratan W. Jadhav, Sheshanath V. Bhosale, Subhajit Bandyopadhyay

**Affiliations:** 1 Indian Institute of Science Education and Research (IISER) Kolkata, Mohanpur, Nadia, WB 741246 India; 2 School of Science, RMIT University, Melbourne, Victoria 3001 Australia; 3 School of Chemical Sciences, Goa University, Taleigao Plateau, Goa 403206 India

**Keywords:** Interlocked molecules, Sensors and biosensors

## Abstract

Stimuli responsive hosts for C_60_ can control its binding and release on demand. A photoswitchable TPE based supramolecular host can encapsulate C_60_ in the *Z*-form with a markedly different visual change in the colour. In addition, the *Z*-1 bound C_60_ has been characterized by various spectroscopic methods and mass spectrometry. Upon exposure to visible light (>490 nm), the host switches to the *E*-form where the structural complementarity with the guest is destroyed as a result of which the C_60_ is disassembled from the host. The results described herein reveals an actionable roadmap to pursue further advances in component self-assembly particularly light-induced association and dissociation of a guest molecule.

## Introduction

The discovery of fullerene is marked as an epoch in the field of organic nanomaterials^1–3^. Since C_60_ is a spherical π framework devoid of any functional groups, construction of its receptor has been a challenge for chemists. A number of host molecules for C_60_ stands as witnesses of ingenuity of molecular architecture^4^. Complementarity of the spherical shape of the buckyball has been achieved by challenging synthesis of molecular bowls, hoops, peapods and several other flexible structures that undergo induced-fit around the molecule. Cycloparaphenylene acetylenes offer supramolecular complexation with fullerene derivatives^5^. Platinum-based molecular cages has also been reported to encapsulate the C_60_^6^_._ Aromatic molecules such as cycloparaphenylene rings encapsulate C_60_ as well as other fullerene derivatives^7,8^. An aza-buckybowl based system have been reported to act as efficient receptors for C_60_ and C_70_ with large association constant^9^. Larger aromatics such as cyclochrysenylenes form molecular “*peapods*”. The complementarity of the convex surface of the buckyball has been achieved by concave bowl-shaped molecules such as corannulenes, sumanenes and even with a expanded rosarin derivative^10–13^. Even a nitrogen-containing buckybowl and its assembly with C_60_ has recently been reported^14^. Non-planar hydrocarbons such as triptycene also form complexes with fullerenes due to geometrical complementarity of their concave shapes to the spherical surface of the C_60_^15^. Fusion of more than one receptor units in a single host molecule displayed enhanced affinity towards the fullerene guest. For instance, a receptor bearing two corannulene units has been reported as a “bucky-catcher” with a high binding constant^16^.

Stimuli-responsive supramolecular host for fullerenes can open up the possibilities for handling the bucky balls and other carbon-based frameworks with superior controls using the stimuli such as pH, electrical or magnetic fields, electrochemical and photonic signals^17–21^. Light is one of the most extensively used stimuli because of its non-invasive nature and easy regulation by the precise focus of its exposure area, and adjustment of its wavelength and intensity^22,23^. Several light-triggered and redox-triggered release of guest molecules have been reported in recent years^24–27^. The selective recognition of C_60_ by a bispyridine ligand with embedded anthracene panels in presence of Ag(I) ion and its light-mediated subsequent release was studied^28^. Reversible host-guest complexation and decomplexation triggered by light can be achieved by the incorporation of photochromic units in the supramolecular systems^29^. Azobenzene is a robust photoresponsive molecule which can exhibit significant structural and chemical modification upon exposure of UV and visible light. These photoswitches have been widely used for the rapid and exact modulation of several biological processes^30–45^.

A theoretical study of host-guest interactions between C_60_ and a photoresponsive group containing nanorings host was investigated and further experimental synthesis of photoresponsive hosts have been predicted^46^. However, reversible binding and release of C_60_ still remain a challenge. C_60_ is known to form stable supramolecular complexes with several compounds having flexible phenyl rings^47^. Crystal structures showing C_60_-tetraphenylethene (TPE) bears the witness of the non-covalent van der Waals interactions between the two species^48^. Recent years, TPE have been widely used due to its abnormal properties i.e. non-luminescent in a solution state and highly emissive upon aggregation and solid state – so called aggregation induced emission (AIE) active chromophore^49,50^.

## Results

**Figure 1:**
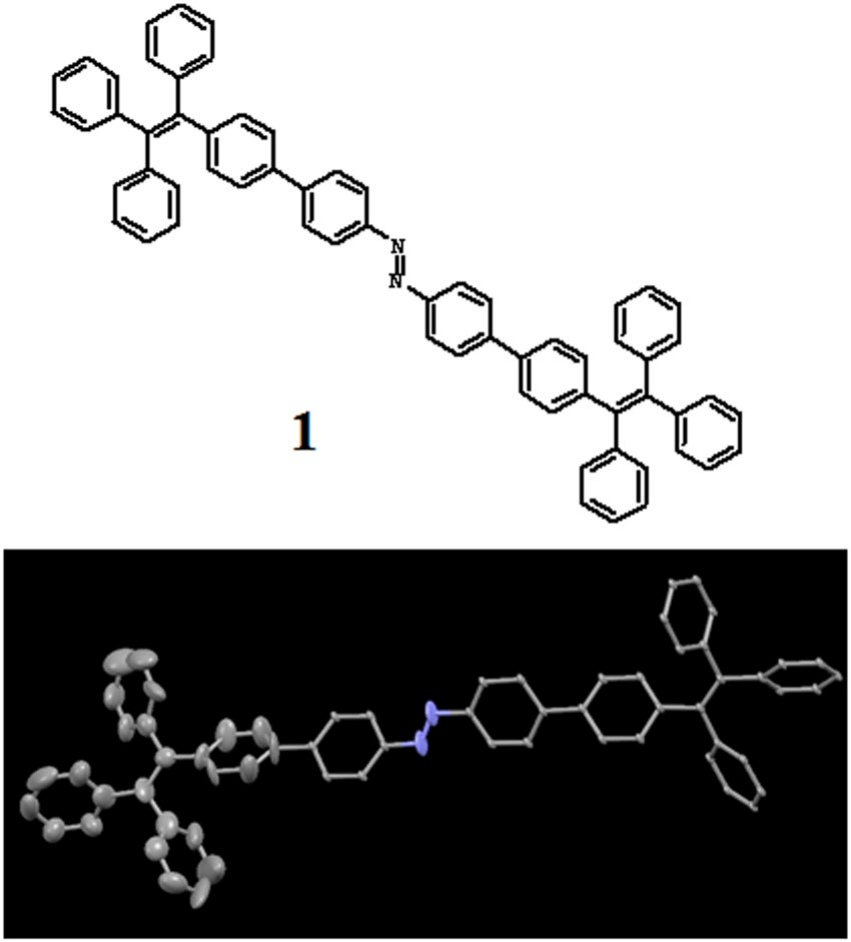
The chemical structure: The structure of the molecule **1** and the crystal structure (50% probability ellipsoids) (CCDC Number 1839412).

Incorporating a photoswitchable azobenzene unit between two TPE moieties, we have synthesized the azobenzene-TPE (**1**, Fig. 1) that contains the elements of supramolecular interactions to bind to C_60_. Binding through supramolecular association is much coveted because it does not perturb the electronic structure of the guest significantly. The azobenzene-TPE photoswitch offers the control of the geometry of the receptor allowed reversible supramolecular interaction with C_60_.

### Synthesis

Azobenzene-TPE **1** was prepared by reacting 1,2-bis(4-bromophenyl)diazene with 3 equiv. of (4-(1,2,2-triphenylvinyl)phenyl)boronic acid in degassed 1,2-dimethoxyethane/2 M Na_2_CO_3_ (3:1, *v/v*) using Pd(PPh_3_)_4_ as a catalyst at 100 °C for 24 h yielded **1** in 69.5% (for details see Supplementary Information).

### Photoisomerization studies of 1

**Figure 2:**
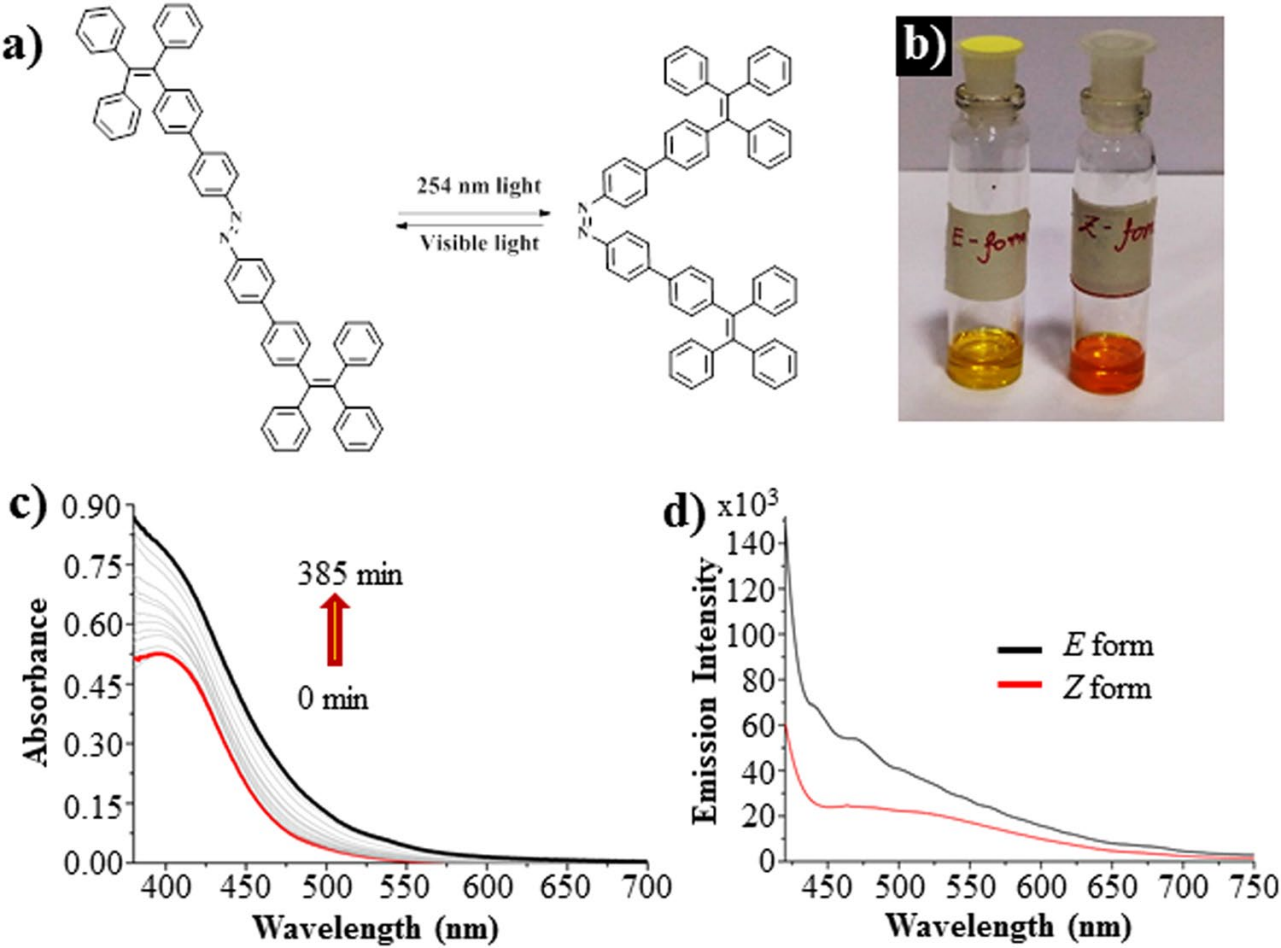
Photoisomerization studies of **1**: (**a**) chemical illustration of *E-Z* isomerization of the molecule **1**. (**b**) Naked eye visualisation of *E-Z* isomers. (**c**) The changes in absorption spectra of the *E* to *Z* isomerization of the molecule **1** (6 µM) in CS_2_. (**d**) Fluorescence spectra of the azobenzene-TPE 1 in both *E* and *Z* forms.

The molecule **1** exhibit *E*-*Z* photochromism where the forward and the backward reactions are fully reversible upon exposure to UV and visible light respectively. Upon irradiation with 254 nm light, the n - π* band of the *E* isomer can be selectively excited leading to the conversion to *Z* isomer as shown in Fig. 2a. This phenomenon can be visualized by the naked eye (Fig. 2b). Under the exposure of 254 nm UV light the band at 400 nm having a molar extinction coefficient (ε) of ~1.04 × 10^5^ M^−1^cm^−1^) becomes broad and the intensity of the band increases with the exposure time. A new broad peak at 550 nm (ε = ~9.8 × 10^3^ M^−1^ cm^−1^) arises due to the n - π* transition of the *Z* isomer which increases in intensity with the 254 nm radiation. The photoisomerization reaction was accompanied by a change in colour that was visually noticeable. The yellow colour of the *E* isomer was transformed to intense orange under isomerization to the *Z* isomer (Fig. 2c) The *E* to *Z* conversion monitored by ^1^H NMR spectroscopy followed a first order kinetics with a rate constant of 6.6 × 10^−2^ m^−1^. The reverse *Z-E* conversion reaction was achieved with visible light with a 400 nm cut off filter. The rate constant for the conversion obtained with the NMR methods was 8.3 × 10^−4^ m^−1^. Furthermore, the fluorescence spectra of *E*−**1** at λ_ex_ = 405 nm displayed a broad band with two peaks at 440 and 470 nm (Φ_F_ = 0.01) and a shoulder at ~550 nm. In the *Z* isomer, the fluorescence intensity was less intense (Φ_F_ = 0.004) with a single band at 480 nm as shown in Fig. 2d.

### NMR studies for isomerization

**Figure 3:**
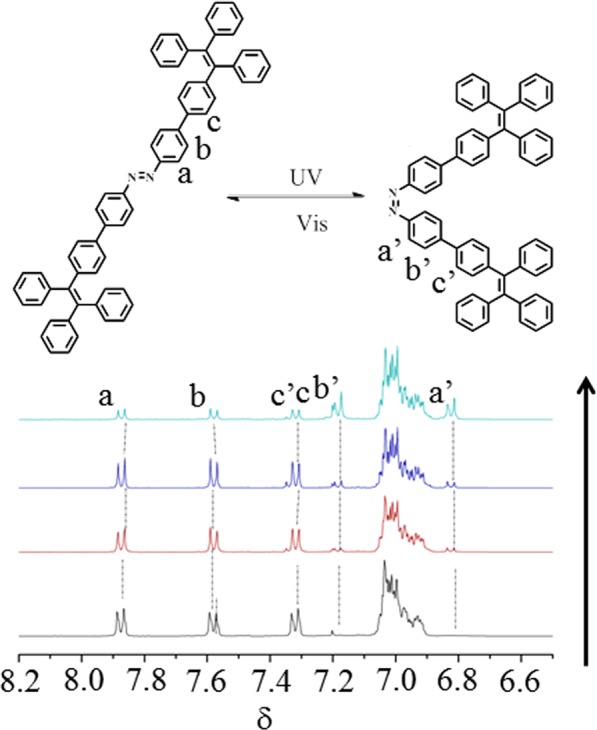
^1^H NMR spectra of *E* to *Z* isomerisation of the molecule **1**.

The *E*-*Z* isomerization of **1** under the exposure of 254 nm light was monitored by ^1^H spectroscopy (Fig. 3) using CS_2_ as the solvent with a small amount of CDCl_3_ for the purpose of locking the instrument. The characteristic signals of the azobenzene moiety of *E*-isomer of **1** were observed at δ 7.87 (H_a_), 7.58 (H_b_), and 7.32 (H_c_). Conversion to the Z-isomer upon exposure to 254 nm UV light triggered an upshifted field shift of the ^1^H resonances and to δ values (a) and (b) protons appear at 7.34 (H_c_’), 7.19 (H_b_’) and 6.83 (H_a_’). The TPE protons appeared as multiplets and their change in the ^1^H NMR spectra was not identifying because of the presence of overlapping multiplets. The composition under the irradiation in the NMR tube corresponded to a ca. 3:1 ratio of the *Z*: *E* isomer.

### Light triggered encapsulation of C_60_ through Photoisomerization

**Figure 4:**
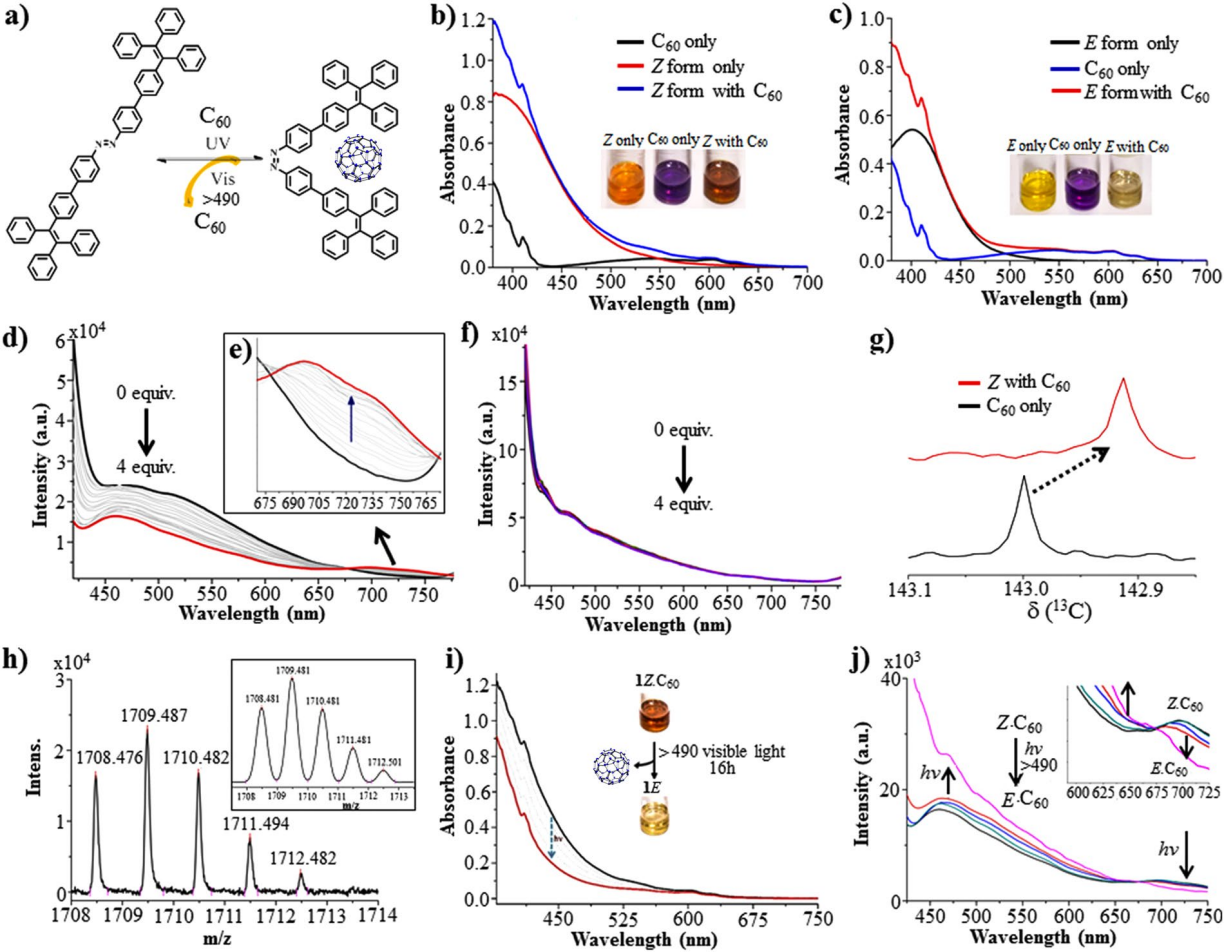
Encapsulation and release of C_60_ by the *Z* form of the molecule. (**a**) structural illustration of host-guest complex and release of guest. (**b**,**c**) Absorbance spectra of the molecule **1** (6 µM) in *Z* forms with C_60_ and *E* form with C_60_ (6 µM) in CS_2_, respectively. (**d**,**e**) Fluorescence spectra of the molecule **1** (6 µM) in *Z* forms with C_60_. (**f**) *E* form with C_60_ (0 to 24 µM) in CS_2_, respectively. (**g)** Changes in ^13^C NMR spectra of C_60_ in presence and in absence of *Z*-isomer of the molecule **1**. (**h**) The MALDI-TOF MS of a sample containing ***Z*-1**.C_60_ indicated the presence of the species perfecly matches well with the calculated isotopic pattern. Changes in (**i**) UV-vis spectra and (**j**) fluorescence spectra of *Z* form of the molecule **1** with C_60_ under the exposure of visible light in CS_2_, which clearly shows release of C_60_ from *Z* form i.e. converting *Z* to *E* isomer with excitation at λ_max_ > 490 nm.

Upon addition of C_60_ to ***Z*-1**, the formation of ***Z*-1.C**_60_ was apparent from the instant change in the absorption spectra. Apart from the obvious change in the visual appearance of the solution ***Z*-1** with **C**_60_ upon mixing (Fig. 4b), the spectrum shows an increase in the peak at 540 nm in presence of C_60_. The increase in absorption band in the region >470 nm indicates the interaction between *Z* form of the molecule and C_60_. The chemical model illustrated in Fig. 4a. In contrary, such changes were not observed with ***E*-1** (Fig. 4c). In fluorescence spectra, (Fig. 4d,e) a new band at 725 nm was observed in the presence of C_60_. In addition, the original fluorescence band of ***Z*-1** along with the shoulder at 520 nm was found to decrease in intensity. A plot of continuous variation (Job’s plot) monitored at 460 nm clearly revealed an association with 1:1 stoichiometry of the host and the guest C_60_ (Supplementary Fig. S1). The binding constant of C_60_ with both ***Z-*1** and for ***E-*1** has been determined from the emission data. As expected the association constant of C_60_ with ***Z-*1** was found to be 4.02 × 10^4^ M^−1^ (Supplementary Fig. S2) and for ***E-*1** with C_60_ the value was less by an order in magnitude with a binding constant of 1.76 × 10^3^ M^−1^. The interaction between *Z*-**1** and C_60_ was also clearly visible with ^13^C NMR spectroscopy (Fig. 4g). Upon addition of C_60_ to ***Z*-1** in CDCl_3_ and CS_2_ (1:10, *v/v*) the peak of the C_60_ at 143.0 ppm underwent an upfield shift to 142.9 ppm because of the encapsulation within the aromatic rings of ***Z*-1**. The change in the ^13^C resonance of the host also provided an insight of the binding site within ***Z*-1** and C_60_ host-guest complex formation, respectively^5,6^. Importantly, The MALDI-TOF MS of a sample containing ***Z*-1.C**_60_ indicated the presence of the species that matches well with the expected isotopic pattern (Fig. 4h).

### Light-triggered release of C_60_ from the *Z* form of the molecule

The *Z* form of the molecule **1** in the presence of C_60_ was exposed to >490 nm visible light, then the broad characteristic charge transfer band in the region >470 nm progressively diminished upon irradiation Fig. 4i). In fluorescence spectra, the band at >700 nm also gradually disappeared (Fig. 4j). The UV-vis spectra indicated near quantitative conversion of the azobenzene unit to the *E* form. This was confirmed by comparison of the spectra (Fig. 4c) with the one obtained upon addition of C_60_ to the pure *E* form of **1**. ^1^H spectroscopic experiments conducted with **1-Z** and C_60_ also pointed towards the formation of a host-guest complex between the two. The facile release of the encapsulated guest molecule (C_60_) takes place in presence of the visible light. The sharp characteristics changes in NMR spectra also prove the encapsulation and release of C_60_ by the *Z* form of the molecule. The ^1^H NMR study was performed with the *Z* form of the molecule **1** in the presence one equivalent of C_60_ in CS_2_. Although the changes in the ^1^H NMR (Supplementary Fig. S3) was less prominent except for the diminished peaks at 7.34, 7.19 and 6.83, the change in the ^13^C NMR was clear. There the peak for the C_60_ at 143.0 shifted upfield to 142.9 ppm (Fig. 4g). This change was reversible under the influence of the visible light. This is anticipated since the C_60_ upon encapsulation by the host TPE groups in *Z* form experiences a shielding effect which causes an upfield shift to the C_60_ nuclei. This change was reversible under exposure to visible light which was also observed earlier with absorption spectroscopy as described earlier.

**Figure 5:**
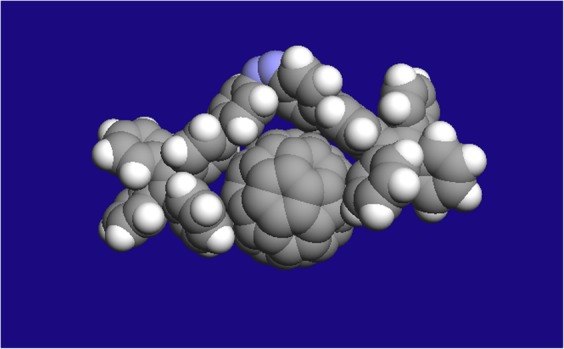
Molecular modeling studies of the encapsulation of C_60_ with the *Z* form of the molecule **1**.

A three dimensional model of the system obtained from computational simulation using DFT calculation at the B3LYP level displayed clear interactions between the receptor and the fullerene. The C_60_ molecule fits in perfectly within the aromatic cavity of the ***Z*-1** form. Several phenyl rings of the molecule puckered around to accommodate the C_60_ with a perfect shape complementarity to form the supramolecular assembly (Fig. 5).

## Discussion

To sum-up, our salient findings of this work are as follows.

1. A TPE-linked azobenzene based photoswitchable molecule has been synthesized and characterized by various spectroscopic techniques.
2. The structural change of the structure upon *E* → *Z* isomerisation of the compound **1** offers the possibility of the formation of a complementary pocket for the accommodation of C_60_ guest molecule.
3. Host-guest interaction with both the *E* and the *Z* isomers of **1** and the C_60_ molecule has been investigated by various spectroscopic techniques.
4. It was observed that the ***Z*-1.C**_60_ association was pronounced compared to the interaction between the ***E*-1 isomer** and **C**_60_.
5. The ***Z*-1.C**_60_ association can be reversed by the exposure of the system with >490 nm light that converts the ***Z*-1 form** to the ***E*-1** form, and thereby weakening the host-guest binding.

Thus this work demonstrates the development of the stimuli responsive host system that can be can be used for light-induced association and dissociation of a C_60_ molecule^51^.

## Supplementary information

Supplementary Information
