# Supplementary material for: Light triggered encapsulation and release of C60 with a photoswitchable TPE-based supramolecular tweezers
Source: Sci Rep. 2019 Jul 4;9:9670. doi: 10.1038/s41598-019-46242-4 (PMC6609608; doi:10.1038/s41598-019-46242-4)
Supplement: Supplementary file 1 — Supplementary Information [file 41598_2019_46242_MOESM1_ESM.docx]

**Supplementary Information**

**Light triggered encapsulation and release of C_60_ with a photoswitchable TPE-based supramolecular tweezers**

Mousumi Samanta,^1†^ Anushri Rananaware,^2†^ Dinesh Nadimetla,^3†^ Sk. Atiur Rahaman,^1^ Monochura Saha,^1^ Ratan W. Jadhav,^2^ Sheshanath V. Bhosale,*^3^ and Subhajit Bandyopadhyay*^1^

^1^Indian Institute of Science Education and Research (IISER) Kolkata, Mohanpur,

Nadia WB 741246 India. ^2^School of Science, RMIT University, Melbourne Victoria

3001 Australia. ^3^School of Chemical Sciences, Goa University, Taleigao Plateau,

Goa-403206, INDIA.





**Figure S1**. The Job’s plot of the molecule **1** in *Z* form in CS_2_.

**Figure S2.** Stern–Volmer plots of the compound in ***E-*1** and **Z-1** form and C_60_ association.


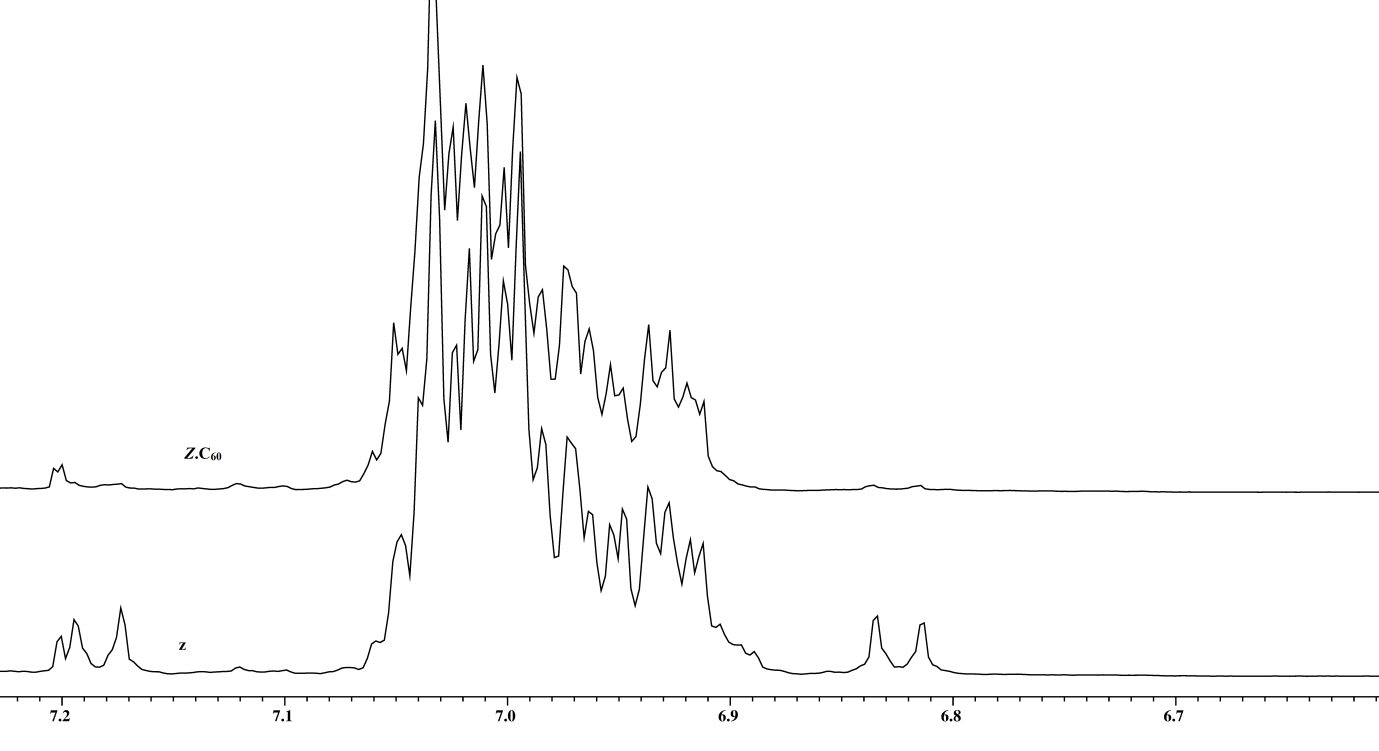


**Figure S3.**^1^H NMR spectra of Z form of the molecule **1**with C_60_ in CDCl_3_/CS_2_ (1: 10).


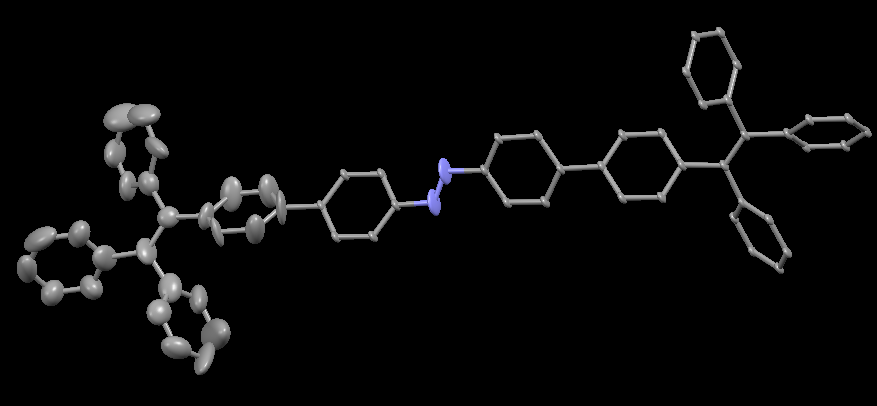


**Figure S4**: Crystal structure of the molecule **1** in *E* form.


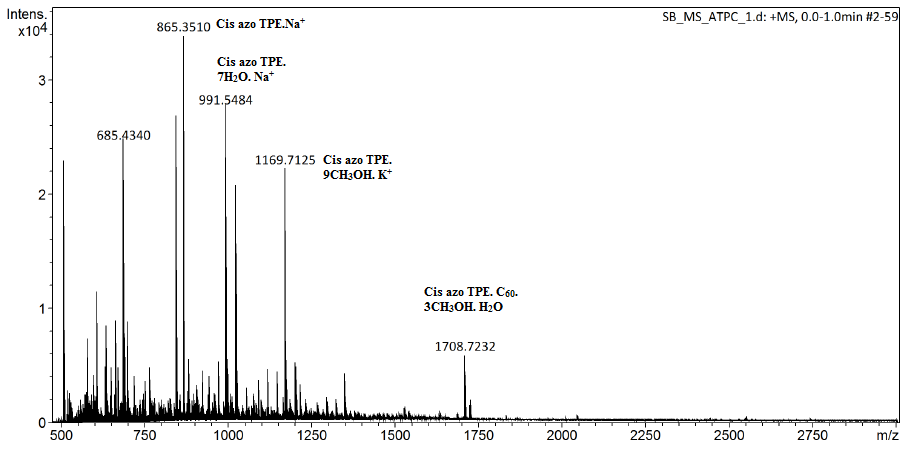


**Figure S5.** ESI-MS of the association of ***Z*.C_60_**.


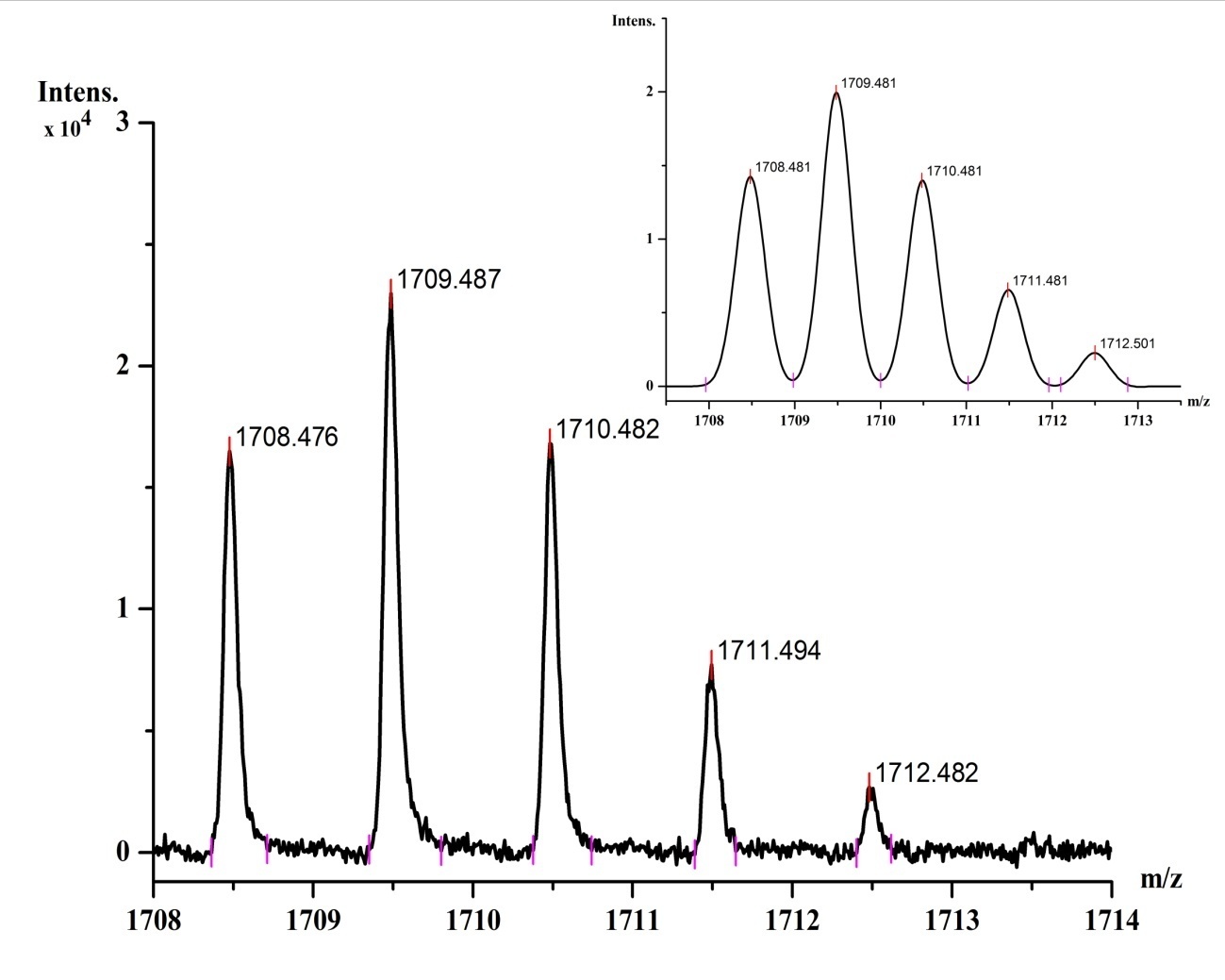


**Figure S6.**The MALDI-TOF MS of the sample containing ***Z*-1.C_60_**showing the expected isotopic pattern.

**Experimental Procedure**

**Scheme S1**. Synthesis of azobenzene-tetraphenylethylene (**Azo-TPE**)

**Step-1: Synthesis of (2-(4-bromophenyl) ethene-1, 1, 2-triyl) tribenzene 4**:This compound was prepared by following a known literature procedure.^S1^

A solution of n-butyl-lithium in hexane (2.5 M; 9.5 mL, 23.79 mmol) was added drop wise to a solution of **2** (4.0 g, 23.79 mmol) in dry THF (175 mL) at 0 °C under nitrogen atmosphere and stirred at same temp for 2 h. Then to it was added a solution of **3** (4.88 g, 18.79 mmol) in THF (30 mL), and resultant was stirred at RT for 10 h. Reaction completion was checked by TLC analysis. Reaction mixture was quenched with aq. NH_4_Cl solution and extracted with DCM (3 × 50 mL). The organic layer was dried over anhydrous MgSO_4_, evaporated on rotary evaporator to give a crude alcohol intermediate. This crude intermediate was further dissolved in toluene (50 mL), and to it was added pTSA (2 g). Resultant was then further reﬂuxed for 16 h. Reaction mixture was cooled to RT, evaporated on rotavapour and crude residue obtained was purified by silica gel chromatography (40−60 nm) to give compound **4** as white solid in 81.6% yield (7.96 g). Analytical data was matched with reported procedure.

**Step-2: Synthesis of (4-(1, 2, 2-triphenylvinyl) phenyl) boronic acid 5**:This compound was prepared by following a known literature procedure.^S2^

To a stirred solution of **4** (4.11 g, 9.997 mmol) in anhydrous THF (80 mL) was added 2.5 M solution of nBuLi in hexane (4.8 mL, 11.99 mmol) at -78 ^o^C and stirred at same temp for 3 h. Then, trimethyl borate (2.28 mL, 19.99 mmol) was added at -78 ^o^C and resultant was allowed to warm at room temperature for 16 h. Reaction completion was checked by TLC analysis. After completion, reaction was quenched by adding conc. HCl and water and then extracted with DCM (3 X 30 mL). Organic layer was separated, dried over MgSO_4_ and evaporated to get crude residue which was then purified by flash column chromatography to afford **5** as an off white solid in 75.6% yield (2.85 g). Analytical data was matched with reported procedure.

**Step -3: Synthesis of 1, 2-bis (4-bromophenyl) diazene 7**:This compound was prepared by following a known literature procedure.^S3^

A finely grinded mixture of KMnO_4_ (2.9 g, 18.38 mmol) and CuSO_4_.5H_2_O (2.93 g, 18.38 mmol) was added to a stirred solution of **6** (1 g, 13.7 mmol) in degassed DCM (80 mL) and resultant mixture was stirred at room temperature for 3-days. After completion, reaction mixture was filtered through celite bed and the residue washed with excess of DCM. Filtrate was evaporated to complete dryness to obtain the crude product. The crude product was purified by column chromatography to afford **7** as an orange solid in 91.9 % yield (1.81 g). Analytical data was matched with the reported values.

**Step-4: Synthesis of 1, 2-bis (4'-(1, 2, 2-triphenylvinyl)-[1, 1'-biphenyl]-4-yl) diazene, 1.**

To a stirred solution of **7** (0.15 g, 0.441 mmol) in degassed 1,2-dimethoxyethane (10 mL) was added **5** (0.497 g, 1.323 mmol), 2M Na_2_CO_3_ solution (3 mL) and resultant was degassed for 15 min using nitrogen atmosphere. Then to it was added Pd(PPh_3_)_4_ (0.025 g, 0.022 mmol) and again degassed for 10 min. Resultant suspension was allowed to heat at 100 ^o^C for 24 h. Reaction completion was confirmed by TLC analysis. After completion, solvent was evaporated to complete dryness and obtained crude was purified by flash column chromatography (40-60 nm) to afford **1** as an orange colour solid in 69.5% yield (0.26 g). ^1^H NMR (300 MHz, CDCl_3_) δ ppm: 7.87 (d, *J* = 8.6 Hz, 4H), 7.58 (d, *J* = 8.6 Hz, 4H), 7.32 (t, *J* = 7.4 Hz, 4H), 7.12 – 6.92 (m, 34H); ^13^C NMR (78 MHz, CDCl_3_) δ ppm: 151.78, 143.60 143.14, 141.41, 140.38, 137.79, 131.93, 131.38, 127.67, 126.58, 126.28, 123.34, 77.68, 76.83, 76.59;MALDI-TOF: for C_64_H_46_N_2_  calculated [M^+^]: 842.3661; found: 843.3721 [M+1]^+^.


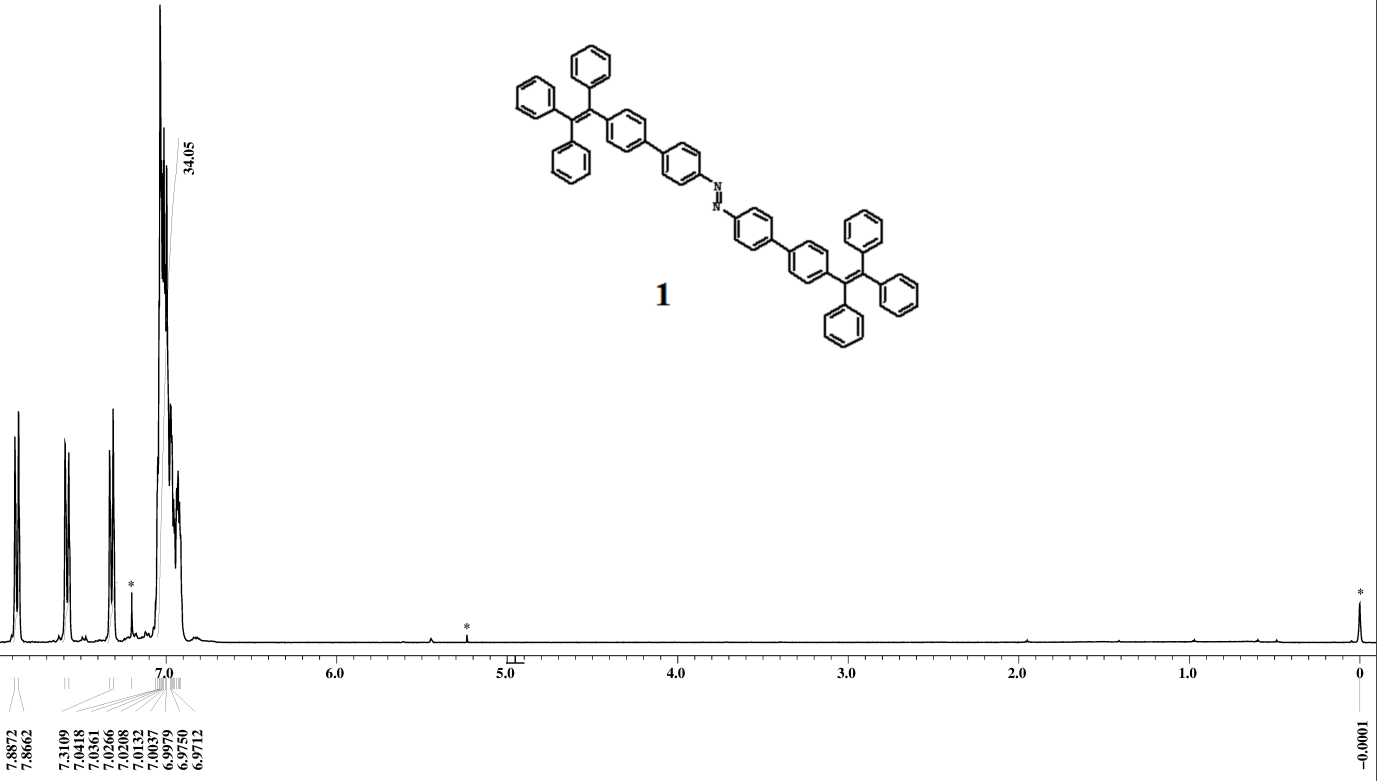


^1^HNMR of **1**: * = solvent peaks


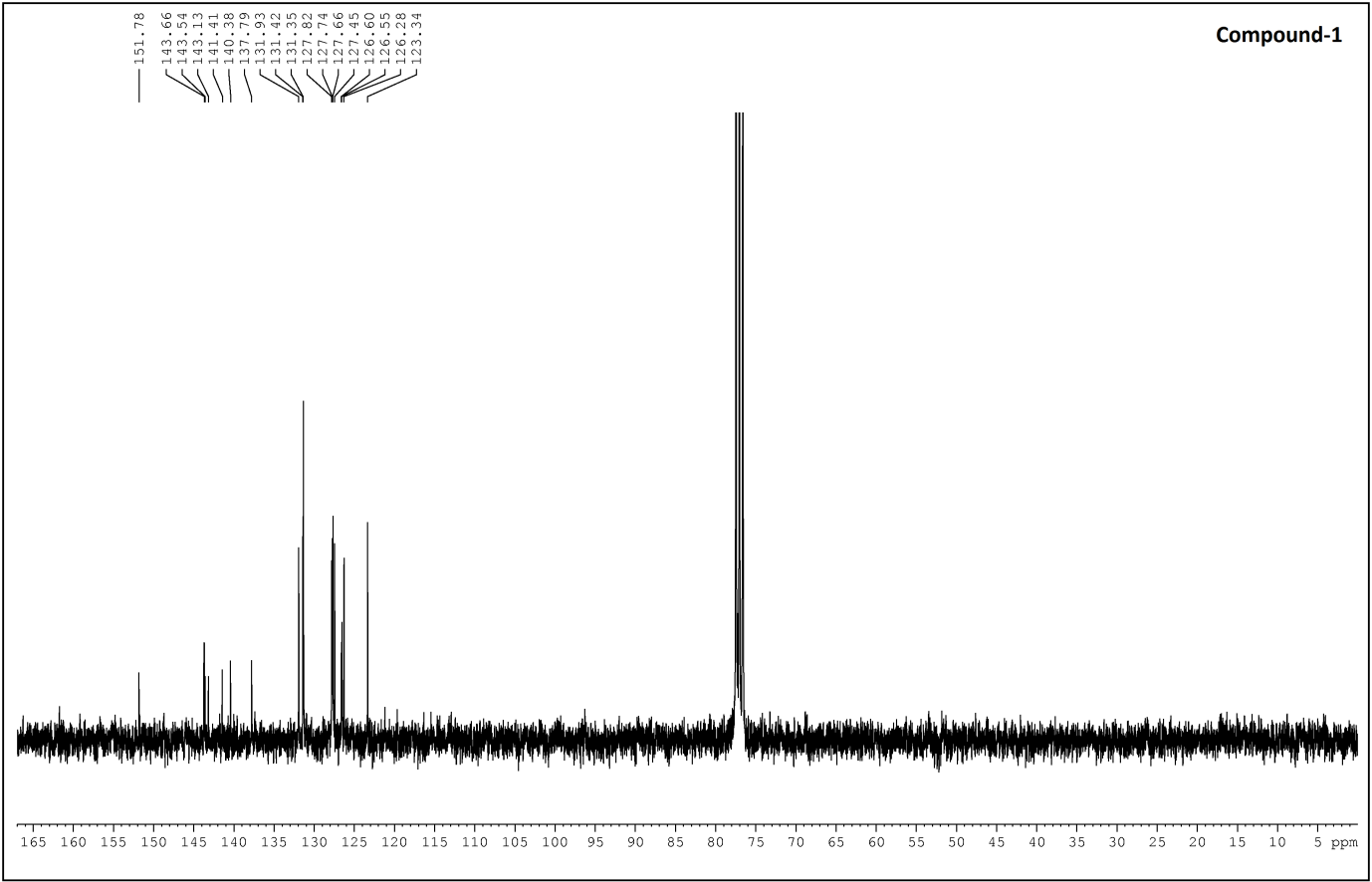


^13^C NMR of **1**


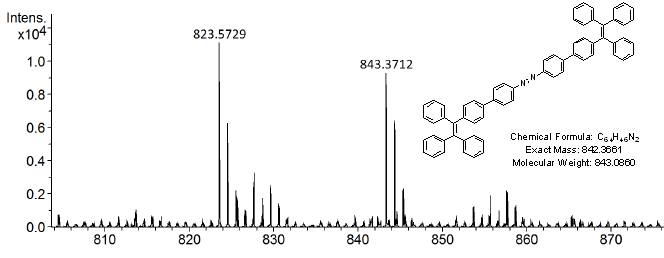


MALDI-TOF HRMS of **1**

**References**

## S1. Yuning Hong, Sijie Chen, Chris Wai Tung Leung, Jacky Wing Yip Lam, Jianzhao Liu, Nai-Wen Tseng, Ryan Tsz Kin Kwok, Yong Yu, Zhengke Wang, and Ben Zhong Tang.FluorogenicZn(II) and Chromogenic Fe(II) Sensors Based on Terpyridine-Substituted Tetraphenylethenes with Aggregation-Induced Emission Characteristics. *ACS Appl. Mater. Interfaces,*2011, *3*, 3411–3418

S2. AnushriRananaware, Rajesh S. Bhosale, Kei Ohkubo, HemlataPatil, Lathe A. Jones, Sam L. Jackson,Shunichi Fukuzumi, Sidhanath V. Bhosale, and Sheshanath V. BhosaleTetraphenylethene-Based Star Shaped Porphyrins: Synthesis, Self-assembly, and Optical and Photophysical Study  *J. Org. Chem.*, **2015**, *80*, 3832–3840.

S3. DörtheGrebel-Koehler, Daojun Liu , Steven De Feyter, Volker Enkelmann, Tanja Weil, Christiaan Engels, Celeste Samyn, Klaus Müllen, and Frans C. De Schryver. Synthesis and Photomodulation of Rigid Polyphenylene Dendrimers with an Azobenzene Core. *Macromolecules*, 2003, 36, 578-590.
